# Development of the Technical Assistance Engagement Scale: a modified Delphi study

**DOI:** 10.1186/s43058-024-00618-4

**Published:** 2024-07-29

**Authors:** Victoria C. Scott, Jasmine Temple, Zara Jillani

**Affiliations:** 1 Department of Psychological Science, 9201 University City Blvd, Charlotte, NC 28223 USA; 2 Department of Sociology, University of Georgia, Athens, GA 30602 USA

**Keywords:** Technical assistance (TA) relationships, TA engagement, Delphi, Measurement development, Formative evaluation

## Abstract

**Background:**

Technical assistance (TA) is a tailored approach to capacity building that is commonly used to support implementation of evidence-based interventions. Despite its widespread applications, measurement tools for assessing critical components of TA are scant. In particular, the field lacks an expert-informed measure for examining relationship quality between TA providers and recipients. TA relationships are central to TA and significantly associated with program implementation outcomes. The current study seeks to address the gap in TA measurement tools by providing a scale for assessing TA relationships.

**Methods:**

We utilized a modified Delphi approach involving two rounds of Delphi surveys and a panel discussion with TA experts to garner feedback and consensus on the domains and items that compose the *TA Engagement Scale*.

**Results:**

TA experts represented various U.S. organizations and TA roles (e.g., provider, recipient, researcher) with 25 respondents in the first survey and 26 respondents in the second survey. The modified Delphi process resulted in a scale composed of six domains and 22 items relevant and important to TA relationships between providers and recipients.

**Conclusion:**

The *TA Engagement Scale* is a formative evaluation tool intended to offer TA providers the ability to identify strengths and areas for growth in the provider-recipient relationship and to communicate about ongoing needs. As a standard measurement tool, it lends a step toward more systematic collection of TA data, the ability to generate a more coherent body of TA evidence, and enables comparisons of TA relationships across settings.

**Supplementary Information:**

The online version contains supplementary material available at 10.1186/s43058-024-00618-4.

Contributions to the literature

- The technical assistance (TA) provider-recipient relationship is central to TA. However, no expert-informed measurement tool exists for assessing TA relationships. This article addresses gaps in TA measurement literature by developing the *TA Engagement Scale*.
- The *TA Engagement Scale* advances TA practice by providing providers and recipients with an expert-informed instrument for monitoring TA engagement quality. The measure increases TA provider ability to make collaborative, data-informed adjustments to TA delivery.
- The scale advances TA by providing a standard measurement tool that aids systematic data collection, formation of more coherent TA evidence, and comparisons of TA relationships across settings.

## Background

Evidence-based interventions and practices (EBIs) are critical for advancing population health and community well-being. However, both uptake and quality implementation of EBIs are a persistent challenge to improved health outcomes globally. A host of contextual factors contribute to implementation-related outcomes, including stakeholder perceptions of the EBI, setting capacity for EBI adoption, and general functioning and climate of the implementation setting [1–3]. A growing body of literature provides evidence that technical assistance improves implementation outcomes [4–8].

Technical Assistance (TA) is a tailored approach to organizational and community capacity building that is chiefly used to support implementation of EBIs [9]. TA involves tailored guidance by a TA specialist (or TA organization) to members of a setting (organization, community) regarding a specific practice area(s) (e.g., needs assessment, program monitoring, etc.). TA delivery frequently entails an assortment of activities (e.g., coaching, consultation, resource sharing; [10]), that vary by recipient need, with direct TA provider-recipient interactions as a hallmark feature. Thus, the relationship between a TA provider and recipient(s) is essential for successful TA [11–13], particularly for intensive models of TA [13]. A provider’s ability to effectively build relationships with recipients is recognized as a core TA competency [14, 15].

In a research synthesis of the TA evidence base, TA relationships (defined broadly as “human encounters between TA providers and recipients”) were discussed in approximately 50% of articles ( [16], p.418). The synthesis affirmed the significance of provider-recipient relationships to TA, noting trust, collaboration, and a strengths-based orientation as most commonly reported relationship attributes. When TA providers establish rapport with recipients, recipients view providers as trusting, respectful, patient, and motivating, underscoring the importance of the recipient-provider relationship [17–19]. Collaborative TA relationships are positively associated with implementation-related outcomes including implementation adherence [19, 20], and high-quality team functioning—a proximal outcome linked to implementation effectiveness [21].

The value of soliciting client feedback on a professional client-provider relationship is an established best practice across a variety of professional fields (e.g. clinical therapy/counseling, coaching, consulting). Most providers have access to robust measurement scales for this purpose. For example, clinicians, consultants, and coaches can select from an assortment of field-tested measures to get patient feedback regarding the working relationship with their clients (e.g., *Therapeutic Bonds Scale* [22], *Consulting Effectiveness Survey* [23], *Executive Coaching Survey* [24]. It is similarly beneficial to assess TA provider-recipient relationships to monitor and improve TA quality. However, TA providers and centers have sparse options for measuring relationship quality; as such, those interested in measuring relational elements of TA have resorted to developing their own measures. For example, Chilenski and colleagues [21] developed a 7-item instrument to measure collaboration—one specific and important feature of TA relationships. The field of TA is in need of an expert-informed measure of TA provider-recipient relationship quality, particularly an instrument that assesses the multiple dimensions of TA relationships. The purpose of the current study was to fill this gap by obtaining subject matter expert input to develop the TA Engagement Scale, which assesses the quality of engagement (relationship) between TA providers and recipients.

## Methods

### Initial scale development

We began with a literature review to determine how previous research in TA and related fields (i.e. clinical therapy/counseling, consulting, coaching) measured provider–client relationship quality. We reviewed measures at the domain and item level and retained the most common domains and associated items across each field. The review generated an initial set of domains and items, which we categorized using the International Coaching Federation (ICF) Framework. We used the ICF framework to organize the domains and items due to similarities between coaching and TA; no equivalent framework exists for TA [25, 26].

Next, we solicited TA subject matter expert (henceforth, experts) input through four meetings. Experts were TA providers and researchers from three organizations identified via convenience sampling. These initial discussions with TA experts focused on the adequacy of the domains (e.g., did domains adequately reflect the most salient features of TA relationship quality?). As we obtained feedback, we revised the pool of domains and items accordingly. Altogether, the literature review and initial expert input led to a preliminary, comprehensive set of 14 domains and 75 items. In what follows, we describe our approach to obtaining expert input and consensus on the domains and items on the TA Engagement Scale using a modified Delphi process. We used a combination of literature review, preliminary expert input, and Delphi process to develop a TA scale that is grounded in TA research and practice.

### Participants

The TA research team involved a university faculty member PI (VS) and two doctoral students (JT, ZJ). We utilized convenience and snowball sampling, wherein TA experts across the United States acquainted with the PI were invited to participate in the Delphi study. Additionally, we asked prospective experts to share contact information for any TA provider, recipient, or researcher who might be interested in participating. Participant inclusion criteria included: i) having a minimum of one year experience with TA, and ii) English speaking. TA providers, researchers, and recipients from six organizations participated in the Delphi process. The TA research team did not participate in the Delphi surveys and were not included in the data analysis.

### Procedures

The Delphi method is a systematic approach for eliciting and aggregating opinion on a topic from a panel of experts [27]. This method has commonly been used to identify the current state of knowledge on a subject, come to a resolution on controversial topics, and develop measurement and indicator tools [28–30]. Typically, respondents engage in several rounds of surveys in which they share feedback on a series of questions. Between rounds of surveys, the researcher analyzes experts’ feedback and consolidates it for the following round so that items with more consensus proceed to the next round of surveys and items with less consensus are eliminated. In prior literature, consensus has been defined as 50–97% or more of subject matter experts in agreement about a subject matter, with a 75% median threshold for defining agreement [29]; however, there is no definitive agreed upon consensus threshold regarding Delphi studies [31].

The TA research team utilized the Accurate Consensus Reporting Document (ACCORD) for a Modified Delphi process [32] to ensure accurate and systematic reporting of the Delphi method. The completed ACCORD document is provided in supplementary material. In the current study, the Delphi consensus building process consisted of two surveys administered to subject matter experts (i.e., TA providers, recipients, and researchers) using Qualtrics, a web-based survey platform. Surveys were not piloted prior to administration to TA Delphi experts, however feedback about the survey content was received during discussions with TA experts (see Initial Scale Development section). The two-round survey design was informed by the research team’s pre-existing work (preliminary feedback from TA experts). Before administration of the first Delphi survey, the team hosted two orientation sessions with prospective experts to introduce the goals of the national study and communicate expectations for participation. An orientation session was recorded and shared with prospective experts who were unable to attend a live session. Each round of the *TA Engagement Scale* survey was emailed to interested experts (described below), who then consented to participate in the study. For Survey Round #1 and #2, we asked experts, “*To what extent are the following domains (and items) and their respective definitions relevant to interactions between TA provider and recipient?*”. Response options ranged from 1 (*not at all relevant*) to 4 (*completely relevant*). The consensus threshold was held at 70%, in which items were retained if 70% or more of experts agreed items were relevant. The 70% consensus threshold was discussed with experts in the orientation session and experts agreed 70% was reasonable and aligned with prior Delphi study methods [29, 33, 34]. After each domain and item, we provided a comment box for experts to leave an open-ended response regarding any comments, suggestions, and concerns related to each domain and item. Additionally, an open-comment box was included at the end of the survey for any other input (e.g. comments about the measure overall, any suggested items, any general questions). The inclusion of open-ended text boxes is a common practice in Delphi surveys [35]. Survey responses were confidential but not anonymous. The first survey was administered over a three week period from August 21st, 2023 to September 8, 2023 and reminder emails were sent on a weekly basis. After we received feedback on survey #1, the TA research team summarized survey results into a report that was shared with the experts. We then held a one hour discussion with the panel of TA experts to discuss their Survey Round #1 feedback and to clarify questions on September 22, 2023.

The second *TA Engagement Scale* survey was administered to the experts over the course of two weeks from October 6, 2023 to October 20th, 2023. Reminder emails were sent on a weekly basis. In Survey Round #2, we again asked experts to rate the relevance of the refined scale domains and items. Additionally, we asked experts to prioritize the items within each domain based on relative importance to the domain reflecting TA relationships. We elevated the consensus threshold to 85% in Survey Round #2 to increase confidence and agreement in survey items and domains. The 15% increase in threshold was established based on practices reported in existing Delphi studies [35, 36]. This national TA Delphi study was approved by the University of North Carolina at Charlotte Institutional Review Board (IRB-23-0463). A study protocol for this research is unregistered.

### Data analysis

Data for Survey Round #1 was analyzed in September of 2023. We followed a four step approach to analyze the quantitative and qualitative data for Round #1 of survey input. First, we used a 70% agreement threshold to determine which domains and items we retained versus removed: if 70% or more respondents indicated that a domain/item was mostly or completely relevant, then the specific domain/item was retained. Domains and items below the 70% threshold were removed. The second and third steps were based on qualitative feedback provided by experts in the open-ended responses. Open-ended responses were analyzed thematically as done in other Delphi studies [28, 33]. In the second step, we assessed if items needed to be relocated to another domain based on expert input (i.e., experts stated in open-ended responses that an item was best represented in another domain). In the third step, we reviewed domains and items that were flagged by experts as redundant (items with similar wording or attributes). If a domain was redundant with another domain, then we merged them and revised the domain definition if needed. If an item shared redundancy with another item, we kept the item with the higher agreement. Lastly, after considering the last three steps, we determined whether an item was retained, removed, or relocated.

Data for Survey Round #2 was analyzed between October to December 2023. In the second round of survey input, we followed a five step approach. First, we used an 85% agreement threshold to strengthen the consensus criteria. If domains or items did not meet the 85% threshold for agreement, then the domain and item was removed. Second, we determined whether the item needed to be relocated based on qualitative feedback. Third, we determined whether the domains or items shared redundancy with other domains and items, respectively; if they did, we merged the domains and retained items with the higher agreement. Fourth, we included a rank ordering system so that respondents could indicate the order of each item’s relative importance from least to most important. The average ranking of these items were used to determine the rank order. The lowest ranked items in a domain were removed. Finally, based on prior steps, we determined whether an item was retained, removed, or relocated.

## Results

**Fig. 1:**
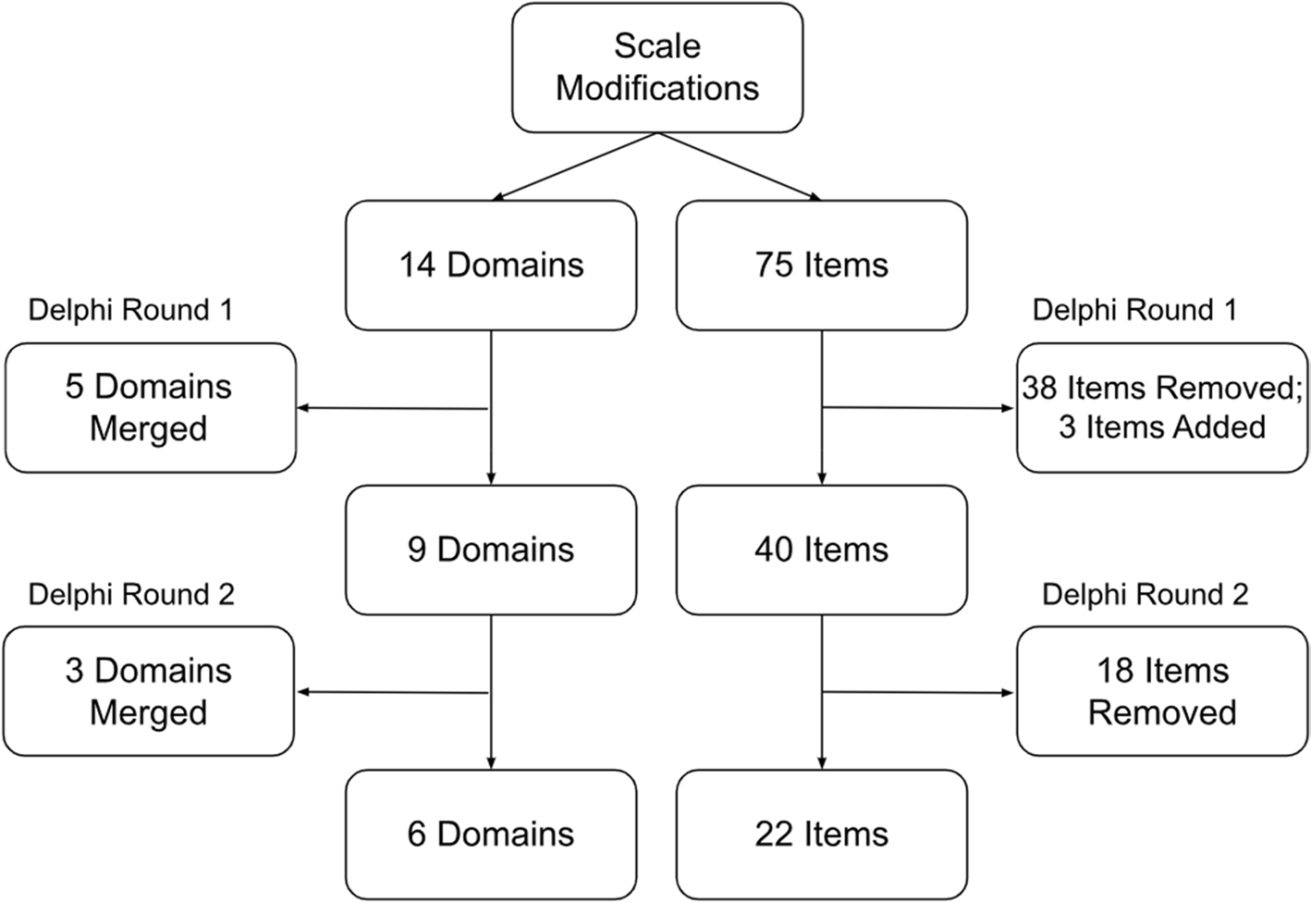
Scale modifications across modified Delphi survey rounds. Note. Decisions made during Delphi panel discussions are reflected under Delphi Round 1

At the beginning of the modified Delphi process, the *TA Engagement Scale* included 75 items classified across 14 domains. After the expert input and consensus process, the final scale was reduced to 22 items across six domains. A summary of scale modifications across the modified Delphi survey rounds is available in Fig. 1. A detailed description of the results for each survey round follows.

### Survey round #1

We sent the first survey to 32 TA experts. Twenty-five experts responded to Survey Round #1 (78% response rate). We asked experts to indicate their role in TA (i.e., provider, recipient, researcher, other), with the option to select multiple roles. Largely, respondents were TA providers (*n* = 24), researchers (*n* = 12), and recipients (*n* = 6). See Table 1 for expert characteristics.

**Table 1:** Delphi participant characteristics

Characteristic	Round #1 n (%)	Round #2 n (%)
***Gender***		
Woman	21 (84%)	22 (85%)
Man	4 (16%)	4 (16%)
Total N	25 (100%)	26 (100%)
***Technical Assistance (TA) Professional Role***^**a**^		
TA Provider	24 (96%)	22 (85%)
TA Researcher	12 (48%)	9 (35%)
TA Recipient	6 (24%)	5 (19%)
Other	2 (8%)	5 (19%)

^a^The TA Professional Role question included ‘select all that apply’ options in which experts could identify as both a TA researcher and provider, for instance. Thus, totals exceed 100%. Some experts, in addition to their main role as a provider, researcher, or recipient, also selected an ‘other’ category, which included roles such as a project officer for a TA center, a funder of TA projects, a TA support person, a funder overseeing TA evaluation contracts, and a grants manager

Survey Round #1 invited feedback on 14 domains and 75 items. All domains reached the 70% consensus threshold. However, qualitative expert feedback indicated redundancy between some domains: Mutual Affirmation and Empathy, Mutual Investment and Collaboration, Responsiveness and Effective Communication, and Focused Facilitation and Accountability domains. In response, we merged these domains and revised their definitions (see Table 2).

**Table 2:** Round #1 domain revisions

Domain	Percentage Agreement: Relevance to TA Relationships	Decision #1 Did the domain meet the relevance threshold? (70%)	Decision #2 Did the domain share redundancy with other domains?	Decision #3 Did the domain language need to be refined?	Final Decision Was the domain retained, removed, or merged?
**Professionalism**	96%	Yes	No	Yes	Retained
**Responsiveness**	96%	Yes	Yes	Yes	Merged with Effective Communication
**Client-Centered**	96%	Yes	Yes	Yes	Removed based on panel discussion
**Ecologically-Minded**	92%	Yes	No	Yes	Retained and changed to Contextually-Minded
**Proactive**	88%	Yes	No	Yes	Retained
**Empathy**	100%	Yes	Yes	Yes	Merged with Mutual Affirmation
**Mutual Affirmation**	80%	Yes	Yes	No	Merged with Empathy
**Trust**	100%	Yes	No	No	Retained
**Collaboration**	92%	Yes	Yes	Yes	Merged with Mutual Investment
**Mutual Investment**	80%	Yes	Yes	Yes	Merged with Collaboration
**Effective Communication**	100%	Yes	Yes	Yes	Merged with Responsiveness
**Focused Facilitation**	80%	Yes	Yes	Yes	Merged with Accountability
**Tailored**	96%	Yes	No	No	Retained
**Accountability**	100%	Yes	Yes	Yes	Merged with Focused Facilitation

At the item-level, we first removed items that did not achieve a 70% consensus threshold (*n* = 4). Then, an additional 34 items were removed due to qualitative feedback indicating the items were redundant with other items, were less clear compared to similar items, and/or were rated lower in comparison to duplicate items. Seven items were relocated as a result of domain-level revisions and qualitative feedback. Finally, three new items were added to better reflect aspects of the Contextually-Minded, Proactive, and Trust domains; experts indicated that the full range of components of the domain were not captured in the original set of items. We ultimately retained 37 of the original 75 items and added three new items for a total of 40 items (see Table 3 for detailed information about the number of domains and items removed, retained, modified, or added in round one).

**Table 3:** Round #1 item revisions

Domain, Item	Percentage Agreement: Relevance to TA Relationships	Decision #1 Did the item meet the relevance threshold? (70%)	Decision #2 Did the item need to be relocated?	Decision #3 Did the item share redundancy with other items?	Final Decision Was the item retained, removed, or relocated?
**Professionalism**					
**Item 1.1. “My TA provider demonstrates professionalism through their conduct.”**	88%	Yes	No	No	Retained
**Item 1.2. “My TA provider upholds the agreements of their services.”**	84%	Yes	No	No	Retained
**Item 1.3. “My TA provider practices with integrity.”**	88%	Yes	No	No	Retained
**Item 1.4. “My TA provider treats me with respect at all times.”**	100%	Yes	No	No	Retained
**Item 1.5. “My TA provider protects my organization’s sensitive information.”**	80%	Yes	No	No	Retained
**Item 1.6. “My TA provider creates a safe space for me to share my organization’s information.”**	92%	Yes	No	No	Retained
**Responsiveness**					
**Item 2.1. “My TA provider is responsive to my expressed needs.”**	100%	Yes	Yes, relocated to Effective Communication	No	Relocated
**Item 2.2. “My TA provider responds to requests for technical assistance in a timely manner.”**	92%	Yes	Yes, relocated to Effective Communication	No	Relocated
**Item 2.3. “My TA provider is easily accessible and available when I need them.”**	92%	Yes	No	Yes	Removed
**Item 2.4. “My TA provider assures me that I can reach out to them anytime.”**	80%	Yes	No	Yes	Removed
**Item 2.5. “I feel I can reach out to my TA provider anytime.”**	88%	Yes	No	Yes	Removed
**Client-Centered**					
**Item 3.1. “My TA provider encourages me to generate my own solutions to challenges.”**	88%	Yes	No	Yes	Removed
**Item 3.2. “My TA provider tries to help me arrive at my own solutions when problems arise, rather than telling me what he/she would do.”**	88%	Yes	No	Yes	Removed
**Item 3.3. “During TA sessions, I am encouraged to share my own views on issues.”**	96%	Yes	No	Yes	Removed
**Item 3.4. “My TA provider values my knowledge and expertise.”**	100%	Yes	No	Yes	Removed
**Item 3.5. “My TA provider encourages me to tackle problems using my own judgment.”**	76%	Yes	No	Yes	Removed
**Item 3.6. “I feel as though my TA provider thinks my opinion is valuable for reaching my goals.”**	80%	Yes	No	Yes	Removed
**Item 3.7. “I feel as though my opinion is valuable to reaching my goals.”**	64%	No	-	-	Removed
**Item 3.8. “My TA provider encourages me to use personal initiative in carrying out my tasks.”**	76%	Yes	No	Yes	Removed
**Ecologically-Minded**					
**Item 4.1. “My TA provider offers suggestions that are appropriate for the setting in which I work.”**	100%	Yes	No	No	Retained
**Item 4.2. “My TA provider is sensitive to relationship issues in my work setting, including power dynamics.”**	96%	Yes	No	No	Retained
**Item 4.3. “My TA provider works to understand the contextual (e.g., political, cultural) aspects of my organization.”**	100%	Yes	No	No	Retained
**Item 4.4. “My TA provider is aware of the conditions and capacities of my organization.”**	80%	Yes	No	Yes	Removed
**Proactive**					
**Item 5.1. “My TA provider communicates emerging issues important to my work and organization.”**	84%	Yes	No	Yes	Removed
**Item 5.2. “My TA provider proactively shares information that can be useful for my work and organization.”**	88%	Yes	No	No	Retained
**Item 5.3. “My TA provider reaches out on their own initiative to provide support for my work and organization.”**	84%	Yes	No	No	Retained
**Empathy**					
**Item 6.1. “I feel my TA provider understands me.”**	92%	Yes	No	Yes	Removed
**Item 6.2. “I feel as though my TA provider and I are on the same wavelength.”**	64%	No	-	-	Removed
**Item 6.3. “My TA provider works to understand my position and perspectives.”**	96%	Yes	Yes, relocated to Mutual Affirmation	No	Relocated
**Item 6.4. “My TA provider works to make sure I feel understood.”**	88%	Yes	Yes	Yes	Removed
**Item 6.5. “At minimum, our interactions are warm and friendly.”**	84%	Yes	Yes	Yes	Removed
**Mutual Affirmation**					
**Item 7.1. “I am able to be honest with my TA provider about my feelings and thoughts on issues.”**	100%	Yes	No	No	Retained
**Item 7.2. “I feel comfortable sharing my skepticism and concerns with my TA provider.”**	100%	Yes	No	No	Retained
**Item 7.3. “My TA provider engages in conversations with an open-mind.”**	100%	Yes	No	No	Retained
**ITEM 7.4. “My TA provider shows their support for me.”**	96%	Yes	No	No	Retained
**Item 7.5. “I feel my TA provider and I support each other in our work together.”**	64%	No	-	-	Removed
**Item 7.6. “My TA provider encourages me to share my thoughts and opinions, no matter how small.”**	84%	Yes	No	Yes	Removed
**Item 7.7. “I feel my ideas are accepted by my TA provider.”**	84%	Yes	No	Yes	Removed
**Item 7.8. “I feel my thoughts and feelings are accepted by my TA provider.”**	84%	Yes	No	Yes	Removed
**Trust**					
**Item 8.1. “I trust my TA provider.”**	96%	Yes	No	No	Retained
**Item 8.2. “I feel I can depend upon my TA provider.”**	92%	Yes	No	No	Retained
**Item 8.3. “My TA provider shows me they are trustworthy.”**	88%	Yes	No	Yes	Removed
**Item 8.4. “My TA provider shows me they are dependable.”**	92%	Yes	No	Yes	Removed
**Collaboration**					
**Item 9.1. “During TA sessions, my TA provider and I are focused on a shared goal(s).”**	92%	Yes	No	No	Retained
**Item 9.2. “My TA provider and I are able to work through disagreements together.”**	84%	Yes	No	Yes	Removed
**Item 9.3. “My TA provider encourages us to work through disagreements and/or conflict together.”**	76%	Yes	No	Yes	Removed
**Item 9.4. “My TA provider and I are able to work through project/organization based obstacles together.”**	92%	Yes	No	No	Retained
**Item 9.5. “My TA provider and I work through project/organization based disagreements together.”**	76%	Yes	No	Yes	Removed
**Item 9.6. “My TA provider and I work collaboratively to generate solutions to challenges.”**	100%	Yes	No	No	Retained
**Item 9.7. “I feel I am working together with my TA provider in a joint effort.”**	80%	Yes	No	Yes	Removed
**ITEM 9.8. “My TA provider encourages us to work together on accomplishing tasks and goals.”**	80%	Yes	No	Yes	Removed
**Mutual Investment**					
**Item 10.1. “I very much want to work through and accomplish my goals with a TA provider.”**	84%	Yes	No	Yes	Removed
**Item 10.2. “I feel that my TA provider and I are both invested and determined in meeting our goals.”**	84%	Yes	No	Yes	Removed
**Item 10.3. “Both my TA provider and I are committed to our working relationship.”**	84%	Yes	Yes, relocated to Collaboration	No	Relocated
**Item 10.4. “I feel my TA provider is committed to our work and goals together.”**	88%	Yes	No	Yes	Removed
**Effective Communication**					
**Item 11.1. “My TA provider asks me questions that show that they are actively listening to me.”**	92%	Yes	No	No	Retained
**Item 11.2. “My TA provider asks me questions that elicit new insight about issues.”**	96%	Yes	No	No	Retained
**Item 11.3. “My TA provider is clear about our shared expectations.”**	88%	Yes	No	Yes	Removed
**Item 11.4. “The things that I am asked to do by my TA provider are presented in a way that I understand.”**	96%	Yes	No	No	Retained
**Item 11.5. “My TA provider is a good communicator.”**	100%	Yes	No	No	Retained
**Item 11.6. “My TA provider’s gestures/mannerisms make them feel approachable.”**	60%	No	-	-	Removed
**Focused Facilitation**					
**Item 12.1. “My TA provider keeps meetings focused and on track.”**	92%	Yes	Yes, relocated to Accountability	No	Relocated
**Item 12.2. “When applicable, I have a clear set of next steps at the end of each TA session.”**	96%	Yes	No	Yes	Removed
**Item 12.3. “My TA provider asks questions that make me think critically.”**	96%	Yes	Yes, relocated to Accountability	No	Relocated
**Item 12.4. “My TA provider has challenged my thoughts and/or actions.”**	84%	Yes	No	Yes	Removed
**Item 12.5. “During TA sessions, we explore different approaches for responding to challenges.”**	100%	Yes	Yes, relocated to Collaboration	No	Relocated
**Tailored**					
**Item 13.1. “What we discuss in TA sessions is highly relevant to my workplace responsibilities.”**	92%	Yes	No	No	Retained
**Item 13.2. “My TA provider offers suggestions that are reasonable to implement given my organization’s capacity.”**	96%	Yes	No	No	Retained
**Item 13.3. “My TA provider shares information and resources that are valuable and relevant to my organization.”**	96%	Yes	No	No	Retained
**Item 13.4. “My TA provider offers me useful feedback and strategies.”**	96%	Yes	No	No	Retained
**Item 13.5. “My TA provider focuses on current assets and resources in my workplace.”**	84%	Yes	No	Yes	Removed
**Accountability**					
**Item 14.1. “My TA provider follows up with me about my commitments.”**	84%	Yes	No	Yes	Removed
**Item 14.2. “My TA provider checks in on my progress related to my commitments.”**	88%	Yes	No	No	Retained
**Item 14.3. “My TA provider identifies clear goals for each session.”**	84%	Yes	No	Yes	Removed
**Item 14.4. “My TA provider guides me to develop clear, achievable action plans for my goals.”**	96%	Yes	No	No	Retained

We shared a report summarizing findings from Survey #1 and invited experts to a one hour panel discussion to review Survey #1 results and to discuss questions emerging from expert feedback. A total of 17 experts attended the panel discussion. As a result of the expert panel discussion, we removed the Client-Centered domain. Experts noted that the Client-Centered domain was more appropriately represented *across* domains rather than separately (that is, nearly every item pertained to the TA provider being client-centered). Additionally, the definition for Professionalism and its items were revised to better measure the issue of privacy in TA relationships.

After completion of Survey Round #1 and the discussion panel, the *TA Engagement Scale* included nine domains and 40 items.

### Survey round #2

We sent the second survey to 32 experts. Twenty-six experts responded to Survey Round #2 (81% response rate). Similar to the first round of respondents, experts were providers (*n* = 22), researchers (*n* = 9), and recipients (*n* = 5), with some respondents indicating more than one TA role.

Survey Round #2 included nine domains and 40 items. All domains reached the 85% inclusion threshold. Based on qualitative expert feedback, we merged Tailored, Contextually-Minded, and Proactive into a single domain, resulting in six domains: Professionalism, Trust, Collaboration, Communication, Tailored, and Accountability (see Table 4).

**Table 4:** Round #2 domain revisions

Domain	Percentage Agreement: Relevance to TA Relationships	Decision #1 Did the domain meet the relevance threshold? (85%)	Decision #2 Did the domain share redundancy with other domains?	Decision #3 Did the domain language need to be refined?	Final Decision Was the domain retained, removed, or merged?
**Professionalism**	92%	Yes	No	No	Retained
**Contextually-Minded**	100%	Yes	Yes	Yes	Merged with Tailored
**Proactive**	89%	Yes	Yes	Yes	Merged with Tailored
**Affirmation**	93%	Yes	Yes	Yes	Merged with Trust
**Trust**	100%	Yes	Yes	Yes	Merged with Affirmation
**Collaboration**	100%	Yes	No	No	Retained
**Effective Communication**	96%	Yes	No	No	Retained; Domain name simplified to Communication
**Tailored**	96%	Yes	Yes	Yes	Merged with Contextually-Minded and Proactive
**Accountability**	92%	Yes	No	No	Retained

At the item-level, Survey Round #2 invited respondents to rank order and rate the importance of the items within each domain. We retained 22 of the 40 items, removing 18 due to failure to meet 85% consensus, item redundancy^1^, or low rank (see Table 5 for additional detail). We shared a report of Survey Round #2 findings with the experts. At the conclusion of the Delphi process, we retained 6 domains and 22 items (see Table 6 for final scale).

**Table 5:** Round #2 item revisions

Domain, Item	Percentage Agreement: Relevance to TA Relationships	Item Importance Ranking	Decision #1 Did the item meet the relevance threshold? (85%)	Decision #2 Did the item need to be relocated?	Decision #3 Did the item share redundancy with other items?	Decision #4 Is the item lower ranked?	Final Decision Was the item retained, removed, or relocated?
**Professionalism**							
**Item 1.1. “My TA provider demonstrates professionalism through their conduct.”**	96%	2	Yes	No	No	No	Retained
**Item 1.2. “My TA provider upholds the agreements of their services (e.g., confidentiality, responsibilities).”**	96%	1	Yes	No	No	No	Retained
**Item 1.3. “My TA provider practices with integrity.”**	85%	4	Yes	No	No	No	Retained
**Item 1.4. “My TA provider treats me with respect at all times.”**	92%	3	Yes	No	No	No	Retained
**Contextually-Minded**							
**Item 4.1. “My TA provider offers suggestions that are appropriate for the setting in which I work.”**	100%	1	Yes	Yes, relocated to Tailored	No	No	Relocated
**Item 4.2. “My TA provider is sensitive to relationship issues in my work setting, including power dynamics.”**	92%	3	Yes	No	Yes	-	Removed
**Item 4.3. “My TA provider works to understand the contextual (e.g., political, cultural) aspects of my organization.”**	100%	2	Yes	Yes, relocated to Tailored	No	No	Relocated
**NEW ITEM. 4.4. "My TA provider encourages me to think about issues holistically**	84%	4	No	-	-	-	Removed
**Proactive**							
**Item 5.2. “My TA provider proactively shares information that can be useful for my work and organization."**	92%	1	Yes	No	Yes	-	Removed
**Item 5.3. “My TA provider reaches out on their own initiative to provide support for my work and organization.”**	88%	2	Yes	No	Yes	-	Removed
**New Item 5.4. “My TA provider checks in with me without waiting for me to reach out.”**	73%	3	No	-	-	-	Removed
**New Item 5.5. “My TA provider stays abreast of issues to anticipate on-going needs of my work and organization.”**	85%	4	Yes	No	No	Yes	Removed
**Affirmation**							
**Item 7.1. “I can be honest with my TA provider about my thoughts and feelings.”**	92%	2	Yes	No	Yes	-	Removed
**Item 7.2. “I am comfortable sharing my skepticism and concerns with my TA provider.”**	100%	1	Yes	Yes, relocated to Trust	No	No	Relocated
**Item 7.3. “My TA provider engages in conversations with an open-mind.”**	92%	3	Yes	No	Yes	-	Removed
**Item 7.4. “My TA provider shows support for me.”**	81%	5	No	-	-	-	Removed
**Item 6.3. “My TA provider works to understand my perspectives.”**	92%	4	Yes	No	No	Yes	Removed
**Trust**							
**Item 8.1. “I trust my TA provider.”**	92%	1	Yes	No	No	No	Retained
**Item 8.2. “I can depend upon my TA provider.”**	96%	3	Yes	No	No	No	Retained
**New Item 8.3. “My TA provider is sincere in our working relationship.”**	81%	4	No	-	-	-	Removed
**New Item 8.4. “I have confidence in my TA provider’s expertise.”**	96%	2	Yes	No	No	No	Retained
**Collaboration**							
**Item 9.1. “My TA provider and I are focused on a shared goal(s).”**	96%	1	Yes	No	No	No	Retained
**Item 9.4. “My TA provider and I can work through project/organization-based obstacles together.”**	96%	3	Yes	No	No	No	Retained
**Item 9.6. “My TA provider and I work collaboratively to generate solutions to challenges.”**	100%	2	Yes	No	No	No	Retained
**Item 12.5. “My TA provider and I explore different approaches for responding to challenges.”**	92%	4	Yes	No	Yes	-	Removed
**Item 10.3. “My TA provider and I are committed to our working relationship.”**	88%	5	Yes	No	No	Yes	Removed
**Effective Communication**							
**Item 11.1. “My TA provider actively listens to me.”**	100%	1	Yes	No	No	No	Retained
**Item 11.2. “My TA provider asks me questions that elicit new insights for me.”**	96%	3	Yes	No	No	No	Retained
**Item 11.4. “I understand the information my TA provider presents to me.”**	96%	4	Yes	No	No	No	Retained
**Item 11.5. “My TA provider is a good communicator.”**	92%	5	Yes	No	No	Yes	Removed
**Item 2.1. “My TA provider is responsive to my expressed needs.”**	100%	2	Yes	No	No	No	Retained
**Item 2.2. “My TA provider responds to requests for technical assistance in a timely manner.”**	100%	6	Yes	No	Yes	-	Removed
**Tailored**							
**Item 13.1. “What we discuss in TA sessions is highly relevant to my workplace responsibilities.”**	92%	1	Yes	No	No	No	Retained
**Item 13.2. “My TA provider offers suggestions that are reasonable to implement given my organization’s capacity.”**	96%	4	Yes	No	No	Yes	Removed
**Item 13.3. “My TA provider shares information and resources that are valuable and relevant to me/my organization.”**	100%	3	Yes	No	No	No	Retained
**Item 13.4. “My TA provider offers me useful feedback and strategies.”**	96%	2	Yes	No	Yes	-	Removed
**Accountability**							
**Item 14.1. “My TA provider checks in on my progress related to my commitments.”**	92%	2	Yes	No	No	No	Retained
**Item 14.4. “My TA provider guides me to develop clear, achievable action plans for my goals.”**	100%	1	Yes	No	No	No	Retained
**Item 12.1. “My TA provider keeps meetings focused and on track.”**	92%	4	Yes	No	No	No	Retained
**Item 12.3. “My TA provider asks questions that make me think critically.”**	100%	3	Yes	No	Yes	-	Removed

**Table 6:** Final domains and items on the TA engagement scale

Domain & Definition	Item	Average Relevance	Average Ranking
**Professionalism:** the extent to which the TA provider upholds integrity and maintains shared agreements about confidentiality and commitments.	My TA provider demonstrates professionalism through their conduct.	96%	2
	My TA provider upholds the agreements of their services (e.g., confidentiality, responsibilities).	96%	1
	My TA provider practices with integrity.	85%	4
	My TA provider treats me with respect at all times.	92%	3
**Trust:** the extent to which the TA provider cultivates a space in which the TA recipient is confident in the dependability, expertise, and openness of the TA provider.	I trust my TA provider.	92%	1^a^
	I can count on my TA provider.	96%	3
	I have confidence in my TA provider’s expertise.	96%	2
	I am comfortable sharing my skepticism and concerns with my TA provider.	100%	1^a^
**Collaboration:** the extent to which TA providers and recipients are committed to working together.	My TA provider and I are focused on a shared goal(s).	96%	1
	My TA provider and I can work through project/organization based obstacles together.	96%	3
	My TA provider and I work collaboratively to generate solutions to challenges.	100%	2
**Communication:** the quality of how information and ideas are exchanged between the TA provider and recipient.	My TA provider actively listens to me.	100%	1
	My TA provider asks me questions that elicit new insights for me.	96%	3
	I understand the information my TA provider presents to me.	96%	4
	My TA provider is responsive to my expressed needs.	100%	2
**Tailored:** the extent to which TA is proactive and responsive to the motivation, capacities, characteristics, and needs of the recipient/recipient organization.	What we discuss in TA sessions is highly relevant to my workplace responsibilities.	92%	1^a^
	My TA provider shares information and resources that are valuable and relevant to me/my organization.	100%	3
	My TA provider offers suggestions that are appropriate for the setting in which I work.	100%	1^a^
	My TA provider works to understand the contextual (e.g., political, cultural, power dynamics) aspects of my organization.	100%	2
**Accountability:** the extent to which the TA provider is clear about goals of the TA sessions and follows-up on issues and commitments to support progress toward desired outcomes.	My TA provider checks in on my progress related to my commitments.	92%	2
	My TA provider guides me to develop clear, achievable action plans for my goals.	100%	1
	My TA provider keeps meetings focused and on track.	92%	4

^a^Some items have the same rank value as others in their respective domains due to item relocation based on qualitative feedback in Survey Round #2. Items were ranked within their original domains and then relocated, meaning some items share rank values

## Discussion

In a seminal paper featuring the support system, Wandersman and colleagues [18] present a model for strengthening the science and practice of implementation support (i.e., Evidence-based System for Innovation Support (EBSIS; [18]). Their work rests on the premise that it is not only important to be evidence-based about community health interventions (e.g., EBIs); it is also important to be evidence-based about the approaches used to support implementation of EBIs, such as TA. Research on the support system is underdeveloped and modest relative to research of the delivery system [37, 38], and tools and methods (e.g. scales, frameworks) to assess TA quality and effectiveness are limited and critically needed [9]. This study contributes to implementation research and practice by providing an expert-informed measurement tool to assess TA relational quality.

We used a modified Delphi approach to develop the *Technical Assistance (TA) Engagement Scale*, a 22-item formative evaluation tool designed to assess TA provider-recipient relationships. Through the Delphi study, we retained six domains: Professionalism, Trust, Collaboration, Communication, Tailored, and Accountability. Five of these domains resemble the relational domains reported in the TA literature synthesis by Katz & Wandersman [18], reinforcing a core set of qualities important to TA relationships: Professionalism (Respect), Trust (Trust), Collaboration (Collaboration), Tailored (Adjusting to Readiness), and Accountability (Roles/Responsibilities). A relational domain that emerged as salient but that was not noted in Katz & Wandersman’s [18] synthesis is Communication. TA experts were consistently high in consensus about the relevance of Communication to TA relationships (96%-100% agreement at the item and domain level), suggesting an area of interpersonal relationships for TA providers to particularly attend to. Aligned with Delphi expert input, effective communication is listed as a key practice for effective TA [10].

The *TA Engagement Scale* critically advances the practice of TA by providing TA providers and recipients with an expert-informed instrument for monitoring TA engagement quality. It enables TA providers and recipients to examine and develop their relationship collaboratively and intentionally. The measure increases TA provider ability to make data-informed, mid-course adjustments to TA delivery. Further, use of the instrument can signal the provider’s high regard for the TA relationship and thereby bolster relationship quality.

In addition to advances in TA practice, the *TA Engagement Scale* can contribute to developments in the science of TA. A standard TA measurement tool is an advancement toward more systematic collection of TA data and is essential to generating a coherent body of evidence. The consistent use of a TA measurement scale across studies will allow TA researchers and evaluators to better compare TA relationships in a variety of settings and to examine correlates of TA relationships with targeted outcomes.

### Use of the TA engagement scale & considerations for research and practice

The *TA Engagement Scale* is intended for administration by TA providers to TA recipients on a periodic basis (e.g. monthly, quarterly, semi-annually) to monitor and improve provider-recipient relationship quality. When the instrument is administered, TA recipients complete the scale by rating the extent to which each scale item is present in their relationship with the TA provider using a 5-point frequency scale (5-Always, 4-Often, 3-Sometimes, 2-Rarely, 1- Never). The TA provider reviews the recipients’ responses to identify relational strengths and areas for improvement. The TA provider is encouraged to discuss the recipients’ feedback with TA colleagues and/or TA recipients. Importantly, this tool is for the purpose of TA relationship monitoring and improvement (formative evaluation). It is not intended as a performance assessment, or as a measure of a TA provider’s performance for a workplace employee evaluation.

We designed the *TA Engagement Scale* with several goals in mind: i) to provide an expert-informed measure that captures multiple dimensions (domains) of TA relationships, ii) to bridge the science and practice of TA through a scale development process involving an in-depth cross-walk of research literature and TA expert input, and iii) to create a user-friendly measure of TA engagement that serves as a practical implementation tool. Given the scale’s relative briefness, TA providers can administer the scale regularly with little time burden on the recipients (< 12 min to complete), making it a practical tool for regularly assessing engagement over time and aligning with calls for more pragmatic approaches to implementation monitoring and tailoring [39, 40].

With modifications, the *TA Engagement Scale* can be used for group TA (i.e., TA involving one or more TA providers and more than one TA recipient). Adaptation of the scale is minor, including revision to the scale instructions to reflect group TA and revising the subject at the scale item-level; for example, revising “My TA provider is responsive to my expressed needs.” to “My TA provider(s) are responsive to our expressed needs.” In collaboration with a national TA center, we have begun to pilot use of the *TA Engagement Scale* in group TA formats. Of note, our modified Delphi study focused primarily on the development of this scale for dyadic provider-recipient relationships. There may be important relationship dynamics in group TA settings uncaptured by the current version of the *TA Engagement Scale*. Research on the use of the *TA Engagement Scale* in group TA is needed to discern if other relational domains beyond the six identified in the scale are important for group TA.

While the *TA Engagement Scale* can be used to assess TA relationship quality across in-person, virtual, and hybrid modes of TA, it may have increasing value for virtual modes of TA. The COVID-19 pandemic catalyzed an acceleration in the provision of remote TA, where TA provider-recipient meetings and trainings shifted from in-person to online modalities to accommodate social distancing mandates and travel restrictions [41, 42]. The increase in reliance on remote TA engenders new questions about how TA provider-recipient relationships are formed and maintained in virtual spaces, including how virtual TA relationships compare to hybrid (in-person/virtua)l and in-person exclusive TA relationships. It is known that there are unique considerations associated with remote TA. For example, virtual settings can present more distractions (e.g. email, social media, multitasking, place-based disruptions) and technological challenges. Specialized preparation by professionals who provide remote services, such as TA providers, is necessary to effectively hold virtual spaces and to engage remote TA recipients [41, 43]. However, the influence of remote TA on TA relationships is less understood. This association merits research as remote TA has become a common practice.

### Study limitations and future directions

Though this scale is informed by TA experts using a multi-stage approach, it has yet to be psychometrically validated. Additional research is needed to establish the measurement’s ability (e.g. test–retest reliability, internal consistency). A next step in the scale development process is to administer the scale in practice and conduct a confirmatory factor analysis to ensure that the items that we have included are measuring the constructs that we intend to measure [44]. We utilized convenience sampling for recruiting the experts whose feedback we used in this study, in which the majority of respondents were TA providers. It is possible that TA recipient opinions and perceptions are underrepresented as the subset of TA recipients was smaller relative to the other groups (TA providers, researchers). However, given that experts were geographically spread and from multiple organizations and backgrounds, we expect that the results of our Delphi process included general and diverse perspectives on what aspects of TA relationships are most central.

The main purpose of the modified Delphi TA study was development of a TA provider-recipient relationship measurement scale. The Delphi study identified six domains highly relevant to TA relationships. We did not seek expert input about the relative importance of these domains over the life course of a TA relationship or across stages of program implementation; the salience of these domains may vary over time. For example, trust may require time to cultivate and thus be positively correlated with relationship length. Collaboration may dwindle over time as TA recipients become more capable and self-reliant. In fact, an association has been reported between the salience of collaboration and implementation stage [16]. Systematic research is needed to better understand the relative importance of each of the six relational domains over the life course of TA engagement.

## Conclusion

The quality of a TA provider-recipient relationship is central to TA and positively associated with program implementation outcomes. Developed through a modified Delphi approach, the *TA Engagement Scale* is a research and expert-informed formative evaluation measurement tool designed to advance the science and practice of TA. It offers TA providers a practitioner-friendly measure for monitoring and improving their relationships with TA recipients. As a standard TA measurement tool, it enables more systematic collection of TA data and thereby, the ability to generate a more coherent body of evidence. The *TA Engagement Scale* can be used to assess relationship quality across multiple (virtual, in-person, and hybrid) TA delivery modalities.

### Supplementary Information

Supplementary Material 1.

## Data Availability

The dataset generated and analyzed during the current study are not publicly available due to confidentiality reasons but are available from the corresponding author upon reasonable request.

## Abbreviations

ACCORD: Accurate Consensus Reporting Document
EBI: Evidence Based Intervention
ICF: International Coaching Federation
RAND: Research and Development Corporation
TA: Technical Assistance

## Footnotes

1 If two items were redundant, we kept the item with the higher rank and agreement.
